# Direct-from-Specimen Detection of Major Carbapenemases by Carbapenem-Resistant K.N.I.V.O. Detection K-Set: Comparative Analysis of Accuracy and Turnaround Time

**DOI:** 10.3390/pathogens15060634

**Published:** 2026-06-15

**Authors:** Basant Mostafa Gabr, Mona Abd El-Aziz Gadallah, Wafaa Abd Elaziz, Sama Metwally, Raghda Gabr Mashaal, Rasha A. Abd Ellatif, Ahmed G. Elkhouly, Hanan Salem, Amira E. Oraiby, Bsant S. Kasem, Sherif Abdelbaky, Reham M. Elkolaly, Marwa S. Taha

**Affiliations:** 1 Department of Medical Microbiology and Immunology, Faculty of Medicine, Tanta University, Tanta 31527, Egypt; basant.mostafa@med.tanta.edu.eg (B.M.G.); mona.gadallah@med.tanta.edu.eg (M.A.E.-A.G.); 2 Department of Biochemistry, Biotechnology Research Institute, National Research Centre, Dokki, Giza 12622, Egypt; we.mohamed@nrc.sci.eg; 3 Department of Internal Medicine, Faculty of Medicine, Tanta University, Tanta 31527, Egyptraghda_mashaal@yahoo.com (R.G.M.); 4 Department of Basic Dental and Medical Sciences, Faculty of Dentistry, Mutah University, Al-Karak 61710, Jordan; dr.rashaabedalazeez@mutah.edu.jo; 5 Department of Anatomy and Embryology, Faculty of Medicine, Tanta University, Tanta 31527, Egypt; 6 Department of Cardiothoracic Surgery, Faculty of Medicine, Tanta University, Tanta 31527, Egypt; ahmed.elkholy@med.tanta.edu.eg; 7 Department of Cardiovascular Medicine, Faculty of Medicine, Tanta University, Tanta 31527, Egypt; 8 Department of Clinical Pathology, Faculty of Medicine, Tanta University, Tanta 31527, Egypt; 9 Department of Tropical Medicine and Infectious Disease, Faculty of Medicine, Tanta University, Tanta 31527, Egypt; smabdelbaky@moh.gov.sa; 10 King Fahd Specialist Hospital, Buradyah 52366, Saudi Arabia; 11 Department of Chest, Faculty of Medicine, Tanta University, Tanta 31527, Egypt; r.elkolaly@med.tanta.edu.eg

**Keywords:** carbapenem-resistant Gram-negative bacteria (CR-GNB), carbapenemase-producing Enterobacterales (CRE), carbapenemase genes, lateral flow immunoassays (LFIA), *bla*_NDM_, *bla*_OXA_, *bla*_IMP_, *bla*_KPC_, *bla*_VIM_

## Abstract

To improve clinical decision-making about Carbapenem-resistant Gram-negative bacteria (CR-GNB) infections and halt the spread of resistant microbes, quicker and less expensive diagnostic techniques are required. Thus, the purpose of this study was to thoroughly evaluate the diagnostic efficiency (sensitivity, specificity, and concordance) of direct-from-specimen multiplex lateral flow immunoassay (LFIA) across diverse raw clinical specimens and pathogen types from critically sick patients. A total of 300 non-duplicate samples were tested to detect CR-GNB. Five major Carbapenemase genes were detected directly from the specimen using carbapenem-resistant K.N.I.V.O. detection K-Set and from culture using culture-enhanced multiplex PCR. Turnaround time (TAT) of each method was calculated. The direct LFIA revealed 100% specificity for NDM, KPC, and IMP enzymes in all tested clinical matrices (blood, urine, and respiratory samples). The study demonstrated 100% sensitivity and specificity with perfect categorical agreement (***κ*** = 1.000) for the *bla*KPC in the *Klebsiella pneumoniae* and for *bla*OXA-48 and *bla*IMP in the *Acinetobacter baumannii*; however, sensitivity of *bla*VIM was significantly diminished across all isolates and samples. TAT decreased significantly (*p* < 0.001) from 30 to 70 h to about 50 min. The tested direct LFIA facilitates the prompt enhancement of lifesaving tailored antibiotic treatment for severe illnesses.

## 1. Introduction

Infections caused by carbapenem-resistant Gram-negative bacteria (CR-GNB) are increasingly reported worldwide and are associated with elevated morbidity and mortality rates, rendering them a significant public health issue [1,2]. The prompt identification of pathogenic microorganisms is essential for effective infection control, appropriate antibiotic management, and improved patient outcomes [2]. Nonetheless, evaluating antibiotic resistance remains a significant challenge, even with advanced techniques such as multiplex PCR tests [3].

Carbapenemase detection in routine clinical microbiology is based on phenotypic assays (e.g., the modified Hodge test, Carba NP, or the EDTA synergy test), immunochromatographic rapid tests, and molecular techniques (e.g., targeted PCR). Whole-genome sequencing (WGS) is becoming a more popular high-resolution reference for genetic characterization and confirmation [4]. Enzyme expression levels and accompanying resistance mechanisms may influence the effectiveness of phenotypic assays, which often require more incubation and may demonstrate limited specificity [5]. PCR provides exceptional accuracy and extensive genetic insights; however, it entails substantial costs, requires specialized equipment, and demands considerable technical expertise, rendering it impractical for routine diagnostics, particularly in resource-limited settings [6].

Considering this backdrop, faster and more affordable diagnostic tools are needed to enhance clinical decision-making and stop the spread of resistant bacteria. Among these emerging technologies are lateral flow immunoassays (LFIAs), which are quick (15–30 min) techniques for identifying clinically significant resistance mechanisms, such as carbapenemases [7]. Additionally, they are typically less expensive than genotypic approaches and need less technical know-how in the absence of costly equipment [8]. Likewise, LFIA systems can identify proteins and are unaffected by false positives when genes are silenced [9]. However, LFIA may yield false-negative results if the target protein is expressed at a level below the detection threshold of the assay, whereas the corresponding gene remains readily detectable by PCR [10].

The predominant approved diagnostic procedures of commercially available LFIA rely heavily on the preceding isolation of pure bacterial colonies, necessitating a required 16-to-24 h overnight agar incubation, which significantly postpones targeted therapeutic interventions [7]. Although recent literature has thoroughly documented the efficacy of LFIAs for the swift identification of CR-GNB directly from positive blood culture bottles [11,12,13], there is a notable lack of data concerning their direct application in a wider range of clinical matrices.

So, this study aimed to systematically assess the diagnostic efficiency (sensitivity, specificity, and concordance) of direct-from-specimen multiplex LFIA, on various raw clinical specimens, such as blood, urine and respiratory matrices, thereby eliminating the traditional overnight culture phase, compared to conventional culture and molecular-based methodologies in critically ill patients. Additionally, it aimed to determine whether this performance is influenced by the microbial genus, specifically comparing its performance between carbapenem-resistant Enterobacterales (CRE) and carbapenem-resistant non-fermenting (CRNF) bacilli. This study also intended to quantify the reduction in turnaround time (TAT) afforded by direct LFIA and its potential impact on early targeted antimicrobial therapy.

## 2. Materials and Methods

### 2.1. Study Design, Location, and Duration

This is a hospital-based cross-sectional study that was carried out in the Medical Microbiology and Immunology Department, the Faculty of Medicine, Tanta University, and the National Research Centre in Egypt during the period of this research from September 2024 to December 2025.

### 2.2. Sample Size Justification

The sample size was determined based on standard formulas for diagnostic accuracy studies. Assuming an expected sensitivity and specificity of approximately 90%, with a 95% confidence level and 5% precision, the calculated minimum sample size was rounded into 300 clinical specimens (100 blood cultures, 100 urine, and 100 respiratory specimens) that were included in the present study. This sample size was consistent with previously published evaluations of lateral flow immunoassays for carbapenemase detection [14].

### 2.3. Ethical Consideration

This research received permission from the Institutional Review Board of Tanta University’s Faculty of Medicine in Egypt (approval code: 36264PR813/8/24).

### 2.4. Inclusion and Exclusion Criteria

This study was conducted on 300 non-duplicate urine and respiratory clinical specimens and positive blood cultures that were obtained from critically ill patients admitted to any intensive care unit (ICU) in Tanta University Hospitals, including surgical, cardiovascular, internal medicine, neurological, cardiothoracic, neonatal and pediatric ICUs. Only eligible specimens showed the presence of predominant GNB on initial Gram-stained smears were included for further examination. Samples with inadequate bulk for parallel arm processing were removed. Respiratory and urinary samples were assessed for quality operating standardized microscopic criteria, those with poor quality were excluded.

For instance, some urine samples exhibited mixed bacterial morphotypes, such as a combination of Gram-positive and Gram-negative bacteria. If these samples also contained a high count of squamous epithelial cells (greater than 10 per low-power field), they were classified as contaminated with commensal periurethral flora and subsequently excluded from the study [15].

Furthermore, respiratory specimens were rejected if they did not meet quality standards through first Gram microscopy that depends mainly on the relative number of squamous epithelial cells (SEC) and polymorphonuclear leukocytes (PMNL) seen microscopically at low-power field (LPF). Microscopic quality evaluation of respiratory specimens was categorized by collecting technique; endotracheal tube (ETT) aspiration or bronchoalveolar lavage (BAL). Aspiration by ETT showed good quality and reflected real infection rather than colonizing flora or contamination, if it showed at least 25 PMNL and less than 10 SEC per LPF. For BAL samples, the complete absence or negligible presence of SECs was necessary to validate the evasion of oropharyngeal bacteria, so guaranteeing that the diagnostic yield accurately reflected the lower respiratory tract environment [16]. In good quality samples, more than 10 microorganisms of the same morphotype at the oil immersion field were considered meaningful [17].

Specimens that exhibited no bacterial growth, produced Gram-positive organisms, or constituted duplicate isolates from the same patient were eliminated from further processing to prevent redundancy and ensure the precision of the prevalence statistics.

### 2.5. Samples Collection and Processing

All samples were gathered under complete aseptic precautions. They were adequately labeled and delivered as soon as possible to the Medical Microbiology and Immunology Department laboratory. A total of 300 distinctive clinical specimens were gathered by a continuing sampling method until the target enrollment was met. The sample set was split into three equal groups for a comprehensive evaluation across different clinical matrices: blood (*n* = 100), urine (*n* = 100), and respiratory secretions (*n* = 100). The respiratory group was additionally categorized to comprise 50 BAL samples and 50 ETT aspirates.

### 2.6. Detection of Carbapenem-Resistant Gram-Negative Bacteria (CR-GNB) by Vitek 2 Compact System

Upon arrival at the laboratory, blood samples were collected in proper blood culture (BC) bottles and incubated in automated blood culture system BC32 (Render Biotech Co., Ltd., Shenzhen, China) till positivity. While urinary and respiratory samples were assessed for quality as mentioned before and Gram-stained smears were prepared and examined microscopically. All enrolled samples were cultured on MacConkey, nutrient, and blood agar plates (HiMedia, Mumbai, India). After overnight incubation at 37 °C, the isolates were identified with further antibiotic sensitivity testing (AST) by VITEK 2 compact system (BioMérieux, Marcy-l’Étoile, France). VITEK^®^ 2 GN cards were used for full identification of Gram-negative bacilli to species level and antibiotic sensitivity was checked by VITEK^®^ 2 AST Cards; AST-N428 for Enterobacterales and AST-N326 for non-fermenters ones to detect CR-GNB.

### 2.7. Culture-Enhanced Multiplex PCR for Detection of the Five Major Carbapenemase Genes

After 18–24 h of incubation of agar plates, DNA was extracted from fresh bacterial colonies by QIAamp DNA Kits (Qiagen, Hilden, Germany) following the manufacturer instructions. Each PCR reaction contained a total volume of 20 µL, consisting of 2 µL of extracted DNA, 10 µL of 2X PCR MasterMix (Thermo Scientific, Lithuania, Waltham, MA, USA), 6 µL of nuclease-free water, and 1 µL of each forward and reverse primer (10 µM) that target the conserved regions of *bla*_KPC_, *bla*_NDM_, *bla*_OXA-48_, *bla*_VIM_, and *bla*_IMP_ genes and are summarized in Supplementary Table S1 [18]. The PCR process was conducted as follows: ten minutes at 94 °C, followed by thirty amplification cycles, each comprising thirty seconds at 94 °C, forty seconds at 52 °C, and fifty seconds at 72 °C, finishing with a final extension of five minutes at 72 °C.

The PCR findings were identified using electrophoresis on 2% agarose gels stained with ethidium bromide. The size of the PCR product was evaluated under UV light using a 100 bp plus DNA ladder (QIAGEN^®^, GelPilot^®^, SYBR^®^, Molecular Probes, Inc., Dreieich, Germany). The discrepancies between the LFA results and the PCR data were examined.

### 2.8. Detection of Carbapenemase Enzymes by Carbapenem-Resistant K.N.I.V.O. Detection K-Set Directly from the Specimen

Simultaneously with the previous modalities, positive blood cultures and four-hours-incubated eligible urinary and respiratory specimens, those samples that shown predominance of GNB in direct Gram-stained smears were subjected for this handling. It is crucial to note that the 4 h short-incubation stage used for lung and urine specimens is an enrichment procedure created especially to reach the necessary bacterial load for the LFIA phenotypic detection threshold. Respiratory samples only were treated with N-acetyl-L-cysteine (NALC) (1:1 ratio) for 15 min to liquefy any mucus that may trap the bacterial cells.

**Figure 1:**
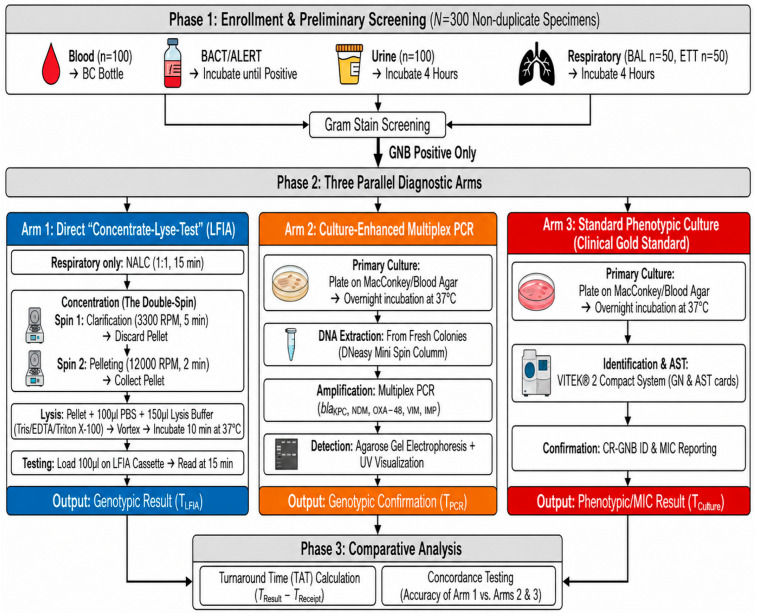
**Schematic representation of the experimental workflow.** The study evaluation was conducted in three sequential phases. Phase 1: Enrollment and preliminary screening, Phase 2: Parallel diagnostic arms, Phase 3: Comparative analysis. ID: Identification, AST: Antimicrobial susceptibility testing, BAL: Bronchoalveolar lavage, BC: Blood culture, CR-GNB: Carbapenem-resistant Gram-negative bacteria, ETT: Endotracheal tube (aspirate), GN: Gram-negative, GNB: Gram-negative bacteria, LFIA: Lateral flow immunoassay, MIC: Minimum inhibitory concentration, PBS: Phosphate-buffered saline, NALC: N-acetyl-L-cysteine (mucolytic agent), RPM: Revolutions per minute. It is important to note that the 4 h short-incubation step utilized for urine and respiratory specimens is an enrichment protocol designed specifically to achieve the requisite bacterial load for the LFIA phenotypic detection threshold. Standard diagnostic procedures include routine primary cultures Following standard clinical microbiology recommendations, parallel Gram stains were carried out on direct, unincubated specimens as soon as they were received by the laboratory.

While testing direct-from-specimen LFIA, the protocol followed three stages “Concentration-Lysis-Testing” as shown in Figure 1. The concentration stage was aligned with the double spin strategy (low-speed clarification followed by high-speed pelleting) using the centrifuge (Thermo Fisher, Loughborough, UK).

Five milliliters from positive aerobic BC bottles and 10 mL from good quality specimens of urine, BAL, or ETT aspirate were transferred into a sterile tube and centrifuged at low speed (3300 RPM for 5 min) to insure clarification of the samples by removal of red blood cells and white blood cells from blood samples, macrophages and undigested mucus chunks from respiratory samples and casts, epithelial cells, and crystals from urine samples. The supernatant is collected in a new sterile tube and the deposit that contained human debris was discarded.

The subsequent high-speed spinning (12,000 RPM for 2 min) was performed to pellet the bacteria in the sample. The supernatant was discarded and the pellet was used for the coming steps.

The lysis stage started by mixing the bacterial pellet with 100 µL of phosphate buffer saline (PBS) and transferred into sterile 1.5 mL Eppendorf containing 150 µL of lab-prepared sterile lysis buffer (50 Mm Tris-HCL plus 50 Mm EDTA plus 0.5% Triton X-100 solution mixed with distilled water and PH was adjusted at 8) and mixed well with vortex.

After 10 min incubation at 37 °C, the testing stage started by adding 100 µL of the mixture into each of the two LFIA cassettes of the carbapenem-resistant K.N.I.V.O. Detection K-Set (Goldstream, Beijing Gold Mountain River Tech Development Co., Ltd., Beijing, China). The test was incubated for 15 min at room temperature as per manufacturer instructions to allow the suspension for crossing the membrane by capillary action and engaged with the immobilized complementary anti-carbapenemase monoclonal antibodies. The results were read visually for control and test lines for the five major carbapenemase enzymes.

### 2.9. Calculation of Turnaround Time (TAT) of Each Protocol

The turnaround time (TAT) was determined for each sample by calculating the difference between the result validation time (when the final finding is officially confirmed = T_2_) and the sample receipt time (when the specimen is first logged into the laboratory information system = T_0_). In this study, T_0_ of blood samples was considered from positivity of BC bottles and after 4 h incubation of urinary and respiratory samples [19].

### 2.10. Statistical Analysis

The data were input into the computer and analyzed using the IBM SPSS software package, version 20.0. IBM Corporation, Armonk, NY, USA. Qualitative data were represented by numerical values and percentages. The results’ significance was assessed at the 5% threshold.

## 3. Results

### 3.1. Distribution of Carbapenem-Resistant Gram-Negative Bacterial Isolates Across Various Clinical Matrices

In the present study, out of 300 eligible specimens, 41.3% (124/300) were GNB. The predominant existence of GNB was in urine samples (44.5%) followed by respiratory samples (40.2%). A total of 60 CR-GNB were isolated across the evaluated clinical matrices. The greatest prevalence of carbapenem resistance was noted in respiratory samples, accounting for 50% of the entire cohort followed by urine samples, including 30% of all CR-GNB isolates. Conversely, blood cultures exhibited the lowest relative frequency of CR-GNB as 20% only, with statistically significant difference across assessed clinical specimen types (Table 1).

Table 2 shows that *Klebsiella pneumoniae* (*K. pneumoniae*) was the predominant CR-GNB, accounting for 38.5% of the overall cohort, with the maximum recovery noted in urine specimens (40%). The non-fermenting pathogens *Acinetobacter baumannii* (*A. baumannii*) and *Pseudomonas aeruginosa* (*P. aeruginosa*) constitute 23.1% and 16.9% of the isolates, respectively, and were significantly present in lower respiratory tract specimens. *Escherichia coli* (*E. coli*) (10.8%) was solely isolated from urinary and respiratory sources, with no instances of bacteremia identified in this group.

### 3.2. Distribution of the Five Major Carbapenemase Genes Across the Isolated Carbapenem-Resistant Gram-Negative Bacteria Using PCR (Reference Standard)

**Figure 2:**
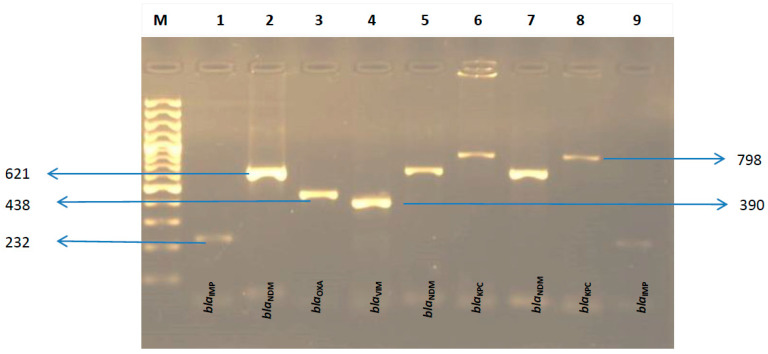
Agarose gel electrophoresis (2%) used for detection of the different PCR products. Lane M: 100 bp plus DNA ladder, lanes 2, 5, and 7 indicate samples with *bla*_NDM_ gene (621 bp), lanes 1 and 9 indicate samples with *bla*_IMP_ gene (232 bp), lanes 4 indicate samples with *bla*_VIM_ gene (390 bp), lanes 6 and 8 indicate samples with *bla*_KPC_ gene (798 bp), and lanes 3 indicates samples with *bla*_OXA_ gene (438 bp).

Multiplex PCR was employed to determine the baseline molecular epidemiology of carbapenemase genes across the isolated CR-GNB (Table 3 and Figure 2). The overall predominant gene was *bla*_NDM_ (58.3%) while the least one was *bla*_VIM_ (36.6%). The non-fermenting group had notable clumping, with *bla*_NDM_ significantly predominating in the *P. aeruginosa* (90.9%) and *A. baumannii* isolates (73%).

### 3.3. Performance Evaluation of Direct-from-Specimen LEIA Across Various CR-GNB

The analytical performance of the direct-from-specimen-LFIA was assessed to ascertain its detection accuracy in Comparison to Multiplex PCR across various biological profiles. Table 4 shows that direct LFIA exhibited considerable target-dependent reliability that varied markedly among bacterial species. The used assay attained optimal detection parameters, exhibiting 100% sensitivity, 100% specificity, and perfect categorical agreement (***κ*** = 1.000) for the *bla*_KPC_ target in the *K. pneumoniae* cohort, as well as for the *bla*_OXA-48_ and *bla*_IMP_ targets in the *A. baumannii* cohort. The LFIA demonstrated exceptional sensitivity (100%) for the *bla*_NDM_ target in *A. baumannii* and *P. aeruginosa*, with overall accuracies of 93.3% and 90.9% for these non-fermenters, respectively.

The direct LFIA effectively identified all real OXA-48 producers in the study group, achieving 100% sensitivity for *K. pneumoniae*, *E. coli*, *A. baumannii*, and *P. aeruginosa*; however, it exhibited five false-positive results in *K. pneumoniae* that significantly diminished its specificity to 64.3% and lowering its inter-rater reliability to moderate agreement (***κ*** = 0.613).

Interestingly, the primary anomaly observed was the widespread diagnostic failure related to the VIM genotype. The inability to identify VIM was widespread throughout both fermenting and non-fermenting groups. The sensitivity of VIM was significantly diminished, peaking at merely 27.3% for *K. pneumoniae* and 25.0% for *P. aeruginosa*, while exhibiting no detection capability (0.0% sensitivity) in the cohorts of *A. baumannii*, *E. coli*, and *Enterobacter cloacae*.

### 3.4. Performance Evaluation of Direct-from-Specimen LFIA Across Various Clinical Matrices

The diagnostic efficacy of this assay was assessed in comparison to the multiplex PCR reference standard in distinctive clinical matrices. In the blood cultures (*n* = 12), the LFIA exhibited significant analytical specificity for most carbapenemase targets. This test demonstrated a specificity and positive predictive value (PPV) of 100% for the *bla*_NDM_, *bla*_KPC_, and *bla*_IMP_ genotypes with perfect categorical agreement between the two tests in the detection of the latest three genes (***κ*** = 0.833–0.800). The sensitivity towards the *bla*_NDM_ target was highest at 85.7%. However, sensitivity for *bla*_OXA-48_ was satisfactory at 80%, a minor decrease in specificity to 85.7% was observed. Nonetheless, a significant diagnostic limitation was identified for the *bla*_VIM_ target, yielding a sensitivity of 0%.

Table 5 shows that the urine specimens (*n* = 18) were like blood cultures as the used assay demonstrated remarkable analytical specificity, with 100% specificity and PPV for the *bla*_NDM_, *bla*_KPC_, and *bla*_IMP_ genotypes with excellent categorical agreement in the detection of *bla*_NDM_ only (***κ*** = 0.880) while considerable agreement for *bla*_KPC_, and *bla*_IMP_ (***κ*** = 0.753–0.675). A slight decrease in specificity was noted for *bla*_OXA-48_ (83.3%), which subsequently reduced its PPV (71.4%). Sensitivity exhibited significant variation among the carbapenemase classes; the test demonstrated optimal performance for the *bla*_NDM_ target, attaining an 85.7% sensitivity and the highest overall diagnostic accuracy of 94.4% while the lowest accuracy was for *bla*_VIM_ target (72.2%). The LFIA exhibited a significant diagnostic limitation in recognizing the *bla*_VIM_ genotype, accurately identifying only 1 of the 6 PCR-confirmed isolates, leading to a clinically inadequate sensitivity of 16.7%.

In the respiratory specimen sub-cohort (*n* = 30), the direct LFIA exhibited strong overall performance for the *bla*_IMP_, *bla*_KPC_, *bla*_NDM_ and genotypes, attaining diagnostic accuracy of 91.4%, 88.6%, and 88.5%, respectively with perfect categorical agreement in the detection of *bla*_IMP_ (***κ*** = 0.824). Like blood and urine samples, *bla*_NDM_ was the most often identified target using PCR (*n* = 22) and had a robust sensitivity of 90.9%. Nonetheless, the respiratory matrix posed significant analytical difficulties for the carbapenemase targets. For instance, sensitivity to *bla*_OXA-48_ target remained elevated at 85.7%; however, specificity dramatically declined to 61.9%, resulting in a poor PPV of 60.0%. In contrast, the *bla*_VIM_ genotype demonstrated a severe deficiency in detection sensitivity (23.1%), accurately detecting only 3 out of the 13 PCR-confirmed cases (Table 5).

### 3.5. Time-to-Result Analysis of Direct-from-Specimen LFIA in Comparison to the Other Two Modalities Across Clinical Specimens

The post-enrichment analytical turnaround times (TAT) for detecting CR-GNB were thoroughly assessed in blood, urine, and respiratory samples using three different diagnostic methods (Table 6). The direct-from-specimen LFIA exhibited a generous and statistically significant decrease in processing time relative to traditional methods across all evaluated specimen types (*p* < 0.001). The direct LFIA for blood samples had a mean TAT of 48.58 ± 10.66 min, significantly surpassing both culture-enhanced PCR (1740.83 ± 217.3 min) and conventional culture with VITEK2 AST (4293.83 ± 1087.6 min). This enhanced performance was notably uniform irrespective of the clinical matrices. Urine and respiratory samples analyzed using direct LFIA produced mean TAT of 52.56 ± 7.7 min and 51.4 ± 9.35 min, respectively. The reference standard culture-enhanced PCR necessitated a median duration exceeding 1725 min (approximately 28.7 h) for respiratory specimens, whereas phenotypic routine culture protocols prolonged detection times to median durations surpassing 3800 min (approximately 63 h) across all sample types as shown in Table 6 and Figure S1.

## 4. Discussion

The synthesis of carbapenemase enzymes is a primary factor in the emergence of CR-GNB and constitutes a substantial worldwide health threat. Given the harmful effects of delayed treatment on patient outcomes, it is imperative to promptly identify the specific resistance mechanism to select successful targeted therapies. Traditional diagnostic methods need extensive bacterial cultures, which can delay clinical results by 72 to 96 h, whereas advanced lateral flow immunoassays like the carbapenem-resistant K.N.I.V.O. Detection K-Set offer a highly accurate alternative, particularly when applied directly on the sample.

In the current study, 41.3% of involved specimens (124/300) showed significant GNB and 30% (60/124) of them were resistant to one or more carbapenems. A comparable resistance pattern has been documented in prior Egyptian studies [22,23]. Elrahem et al. and Lathakumari et al. observed reduced prevalence of carbapenem resistance to be 20% [24] and 24% [25], respectively, whereas Alkaik et al. reported a heightened prevalence to 78.2% [26]. This global variability in reported prevalence of resistance to carbapenems is influenced by multiple factors, namely regional endemicity, differences in the clinical severity of the investigated populations, and the evolving sensitivity of the diagnostic tests used.

The predominant existence of CR-GNB, in this study, was retrieved from respiratory samples collectively (50%), urine samples (30%) then blood samples (20%). In alignment with our findings, an additional study indicated that among 239 cases of CR-GNB, 57.32% were derived from sputum specimens, 26.78% from urine specimens, and merely 4.18% from blood samples [27]. In contrast, the healthcare-associated infection surveillance system in Egypt that investigates the prevalence of CRE in 72 hospitals across 25 governorates, from 2011 to 2017, reported that blood was the most common specimen type for CRE cases compared to other specimen types [28].

*Klebsiella pneumoniae* were the most prevalent CR-GNB isolates from whole collected specimens (38.5%), followed by *A. baumannii* (23.1%) and *P. aeruginosa* (16%). The predominance of *K. pneumoniae* among CR-GNB across clinical samples was reported by many studies throughout the world [22,23,24,25,26,27,28]. This global dominance of *K. pneumoniae* within the CR-GNB cohort is primarily due to its remarkable genomic malleability, enabling the swift acquisition of carbapenemase-encoding plasmids, alongside the extensive spread of highly adapted, biofilm-producing epidemic clones that can colonize various anatomical sites and medical devices [29]. The significant presence of non-fermenting pathogens *A. baumannii* and *P. aeruginosa* in lower respiratory tract specimens was logical due to their dependence on oxygen for metabolism; they demonstrate an inherent biological affinity for the oxygen-rich environment of the lower respiratory tract. This becomes the lungs their favored anatomical habitat for colonization and infection. Moreover, they exhibit strong biofilm-forming abilities on abiotic surfaces, enabling them to densely colonize endotracheal tubes, evade host mucociliary clearance, and establish persistent lower airway infections, as evidenced by the specimen distribution in this cohort [30,31].

The predominant gene among isolated CR-GNB in this work was *bla*_NDM_ (58.3%), succeeded by *bla*_IMP_ (48.3%) and *bla*_OXA-48_ (41.6%), followed by *bla*_KPC_ (40%) and *bla*_VIM_ (36.6%). Alternative studies yielded various outcomes. For instance, Lathakumari et al. revealed the predominance of *bla*_NDM_ and bla_VIM_ in all tested CR-GNB [25] while Alkaik et al. identified *bla*_OXA-48_ as the most prevalent carbapenemase gene, accounting for 70.8% (*n* = 46), followed by *bla*_NDM_ at 15.4% (*n* = 10) [26]. Current epidemiological surveillance in Egyptian healthcare facilities reveals that *bla*_NDM_ and *bla*_OXA-48_ predominantly characterize the carbapenemase genetic landscape in Gram-negative isolates, especially among Enterobacterales. In contrast, the *bla*_VIM_ genotype demonstrates an exceptionally low endemicity in this location [22,32,33,34]. For example, study done across four Egyptian hospitals found that *bla*_NDM_ (80.5%) was the most prevalent, followed by *bla*_VIM_ (36.4%), but it revealed a lower prevalence of *bla*_KPC_ (28.6%), *bla*_OXA-48_ (26%), and *bla*_IMP_ (6.5%) compared to our findings [32].

Furthermore, Badran et al. noticed that the most prevalent genes were *bla*_NDM_ (84.4%), then *bla*_OXA-48_ (73.3%), *bla*_KPC_ (13.3%), *bla*_IMP_ (2.2%); however, *bla*_VIM_ gene was not detected at all [22].

The diverse distribution of the five principal carbapenemase genes in worldwide literature represents the ongoing antibiotic resistance challenge. The prevalence of certain targets is significantly influenced by regional endemicity and the geographic evolutionary centers of mobile genetic elements and is inherently influenced by the species composition of the sample group. For instance, there exists a robust link between OXA-48 enzymes and Enterobacterales, alongside a predilection of metallo-beta-lactamases for non-fermenting bacilli [35].

While testing the diagnostic performance of the direct-from-specimen LFIA across various CR-GNB, it exhibited significant variability among bacterial species. This assay attained ideal diagnostic parameters (100% sensitivity and 100% specificity) for identifying *bla*_KPC_ in *K. pneumoniae*, together with *bla*_OXA-48_ and *bla*_IMP_ in *A. baumannii*. The swift and precise detection of the *bla*_NDM_ target in both *A. baumannii* and *P. aeruginosa* (exceeding 90% accuracy) points out the LFIA’s significant clinical value in critical care environments. Rapid identification of *bla*_NDM_ and *bla*_KPC_ producers directly from raw clinical samples facilitates prompt, targeted de-escalation of empirical medication, eliminating the need for conventional 24 h subcultures and greatly enhancing antimicrobial stewardship. Recently, another study assessing LFIA performance revealed that the overall diagnostic accuracy was predominantly higher for *K. pneumoniae* than for non-fermenting bacilli such as *P. aeruginosa* [36].

Moreover, the false-positive OXA-48 enzyme signals identified solely in *K. pneumoniae* (5 false positive/25 isolates) indicate possible cross-reactivity; this assay may identify hyper-produced endogenous beta-lactamases such as bla_SHV_, or certain non-carbapenemase OXA-48 variants (e.g., OXA-163) that are prevalent in *Klebsiella* species [37]. Clinically, misinterpreting these false-positive results as true carbapenemase production could lead to the unwarranted escalation of antimicrobial therapy to costly, last-line reserve agents (such as ceftazidime-avibactam or cefiderocol). This not only exposes the patient to unnecessary drug toxicity but also risks driving further antimicrobial resistance, highlighting that positive OXA-48 signals in *Klebsiella* require clinical wisdom and demand genotypic verification.

Furthermore, Lee et al. assessed the impact of inoculum size on the efficacy of two distinct LFIA employing 27 Enterobacterales. They noticed that the variation in inoculum sizes may have influenced the test results, leading to false-positive NDM and OXA-48-like outcomes with larger inocula, despite the use of a 1 μL loop to obtain the isolate for testing; thus, they suggest further investigations are necessary to readjust the inoculum size while using commercially available LFIA [38].

The persistent false-negative outcomes for the *bla*_VIM_ genotype in *K. pneumoniae*, *P. aeruginosa*, and *A. baumannii* underscore a significant diagnostic constraint. The sensitivity of *bla*_VIM_ peaked at merely 27.3% in *K. pneumoniae* and 25.0% in *P. aeruginosa*, while it entirely failed to detect the target in *A. baumannii*, *E. coli*, and *Enterobacter cloacae*. This widespread failure suggests that the VIM-specific antibodies employed on the test strip probably possess inadequate affinity or avidity for the specific *bla*_VIM_ allelic variations present in this patient population. In contrast, another extensive multicenter clinical study performed in the USA reported almost 100% analytical sensitivity for the VIM target utilizing the traditional bacterial colonies-based NG-Test Carba 5, unlike the K.N.I.V.O. Detection K-Set directly used in this study [39].

Moreover, the current work tested the diagnostic performance of the direct-from-specimen LFIA across distinctive clinical matrices. Throughout the whole involved clinical sample types, the LFIA assay demonstrated 100% specificity and PPV for the NDM, KPC, and IMP, signifying no false-positive detections for these enzymes in the involved matrices. This supports the reliability of this assay, so a positive result for these genes in blood, urine or respiratory samples can be trusted by the doctor. Furthermore, the highest peak sensitivity exhibited by this assay was for the *bla*_NDM_ target among all targets across all matrices (85.7% in blood and urine and 90.9%in respiratory samples). This highlights the clinical efficacy of this test in identification of the *bla*_NDM_ genotype. The significant therapeutic constraints linked to NDM-producing pathogens necessitate the rapid and accurate identification of this metallo-beta-lactamase from clinical samples, which is essential for optimizing targeted antimicrobial treatment and implementing rigorous infection control measures well. For instance, the NDM-producing pathogens can degrade nearly all beta-lactam antibiotics and, in contrast to KPC, is not suppressed by existing common combinations such as ceftazidime-avibactam alone. If a clinician identifies a patient with NDM-producing pathogen within 2 h using the LFIA assay directly from the sample, rather than pending culture results, they can promptly initiate specialized salvage therapy such as Ceftazidime-avibactam combined with Aztreonam, or Cefiderocol. This rapid response protects lives especially in critically ill patients with bacteremia or pneumonia.

This outstanding performance is well-supported by current work assessing multiplex LFIAs in which they assessed the NG-Test Carba 5 using bacterial isolates and reported a sensitivity of 100% for detection of KPC and NDM enzymes [40]. Likewise, when directly applied to positive blood culture broths, Stokes et al. demonstrated exceptional overall sensitivities for these key targets [13]. This collective agreement affirms that lateral flow technology is exceptionally reliable for identifying KPC and NDM producers, irrespective of whether the assay is utilized on cultivated colonies, positive BC broths, or directly on raw clinical specimens.

Despite the recorded high sensitivity in detection of *bla*_NDM_, there was 14% false-negative rate still exists, so the LFIA is proficient for the immediate identification of this genotype; nevertheless, a negative result must be corroborated by PCR or culture if the patient is critically unwell.

Regarding the ability of the LFIA to detect *bla*_OXA-48_ target, variations were found in sensitivity and specificity across clinical matrices. In urine samples, the diagnostic assessment of the assay produced a balanced analytical profile, with an overall sensitivity and specificity of 83.0%. The test’s primary clinical value was demonstrated by its elevated NPV of 90.9%. In a critical care environment, this strong NPV establishes the test as an exceptionally effective “rule-out” instrument, granting doctors early assurance to safely defer targeted, broad-spectrum antibiotic treatments while awaiting conclusive culture results.

In respiratory samples, sensitivity to *bla*_OXA-48_ target remained elevated at 85.7%; however, specificity dramatically declined to 61.9%, resulting in a poor PPV of 60.0%. The elevated incidence of false-positive LFIA results indicates possible matrix interference, likely restricting non-specific antibody binding attributed to the high viscosity, mucin concentration, or biofilm presence characteristic of lower respiratory tract secretions.

Recent evidence employing direct clinical matrices closely resembles our unusual findings. García et al. assessed the K.N.I.V.O. IC test directly from positive blood cultures and showed a similarly alarming decrease in specificity to 80.8%. They specifically identified 13 false-positive bands, primarily associated with *bla*_OXA-48_ and *bla*_NDM_, which were predominantly concentrated in clinical samples of *E. coli* and *K. pneumoniae* [11]. This strongly indicates that the application of LFIAs directly on complex biological matrices, as opposed to pure isolates, may lead to matrix interference or the activity of endogenous enzymes in Enterobacterales, resulting in non-specific binding and requiring confirmatory molecular algorithms.

On the other hand, the lowest sensitivity was detected for the *bla*_VIM_ genotypes across all specimens (0% in blood, 16.7% in urine and 23.1%); this means that the LFIA was unable to detect any of the three PCR-confirmed VIM-producing isolates in blood and VIM enzyme is consistently missed across different involved clinical matrices. This may result from diminished protein expression of *bla*_VIM_ in these samples.

## 5. Conclusions and Limitations

The current study documented a significant decrease (*p* < 0.001) in TAT (from 30 to 70 h to roughly 50 min). This direct LFIA converts carbapenemase detection from a retrospective measure into a real-time intervention, facilitating the prompt enhancement of life-saving targeted antimicrobial therapy for critical infections such as bacteremia and ventilator-associated pneumonia. These statistical findings are strongly aligned with another work [41].

The introduction of a direct-from-specimen LFIA signifies a crucial transformation in the swift identification of carbapenem-resistant infections. The test showed remarkable diagnostic precision and dependability for prevalent targets, particularly *bla*_KPC_ and *bla*_NDM_, facilitating the swift enhancement of targeted antimicrobial treatments. Nonetheless, the LFIA is not uniformly flawless in all clinical matrices. The distinct, species-specific *bla*_OXA-48_ false-positive signals identified in *K. pneumoniae*, along with the systemic inability to detect regionally endemic *bla*_VIM_ variants, highlight a significant limitation: the efficacy of LFIA is inherently connected to the endemic enzymatic background of the host bacterium and the local molecular epidemiology. Therefore, although the direct LFIA is a significantly disruptive and efficient frontline triage instrument, it should be complemented with region-specific molecular confirmatory tests to guarantee unwavering diagnostic safety.

## Figures and Tables

**Table 1:** Distribution of cultured pathogens and their carbapenem-resistance profiles across clinical specimen types.

Standard Culture-Based Results	Number of Isolates *n*/Total (%)	Blood *n*/Total (%)	Urine *n*/Total (%)	Respiratory *n*/Total (%)		*p*-Value
				ETT	BAL	
Gram-negative Bacteria	124/300 (41.3%)	20/124 (16.1%)	54/124 (44.5%)	25/124 (20.1%)	25/124 (20.1%)	<0.01
- Carbapenem-Resistant (CR-GNB) *	60/124 (48.3%)	12/60 (20%)	18/60 (30%)	15/60 (25%)	15/60 (25%)	
- Carbapenem-Susceptible (CS-GNB)	64/124 (51.6%)	8/64 (12.5%)	36/64 (56.3%)	10/64 (15.6%)	10/64 (15.6%)	
Gram-Positive Bacteria	53/300 (17.7%)	23/53 (43.3%)	8/53 (15.1%)	7/53 (13.2%)	15/53 (28.3%)	
*Candida* spp.	12/300 (4%)	5/12 (41.7%)	5/12 (41.7%)	2/12 (16.7%)	0/12 (0%)	
No Growth/Non-Significant Flora	111/300 (37%)	52/111 (46.8%)	33/111 (29.7%)	16/111 (14.4%)	10/111 (9%)	
Total	300/300 (100%)	100/300 (33.3%)	100/300 (33.3%)	50/300 (16.7%)	50/300 (16.7%)	

ETT: endotracheal tube, BAL: bronchoalveolar lavage, CS-GNB: carbapenem-resistant Gram-negative bacteria, CR-GNB: carbapenem-resistant Gram-negative bacteria, * Gram-negative bacterium that demonstrates non-susceptibility to any single carbapenem (e.g., imipenem, meropenem, or ertapenem) according to standard clinical laboratory standard institute (CLSI) breakpoints [20].

**Table 2:** Prevalence of isolated carbapenem-resistant Gram-negative pathogens across clinical matrices.

Carbapenem-Resistant Gram-Negative Bacteria	Number of Isolates (%)	Blood Samples	Urine Samples	Respiratory Samples		*p*-Value
				ETT	BAL	
Fermenters	34 (56.6%)					0.423
- *Klebsiella pneumoniae*	25 (38.5%)	5 (20%)	10 (40%)	5 (20%)	5 (20%)	
- *Escherichia coli*	7 (10.8%)	0 (0%)	4 (57.1%)	2 (28.6%)	1 (14.3%)	
- *Enterobacter cloacae*	2 (3.1%)	0 (0%)	0 (0%)	1 (50%)	1 (50%)	
Non-fermenters	26 (43.3%)					
- *Acinetobacter baumannii*	15 (23.1%)	5 (33.3%)	1 (6.7%)	4 (26.7%)	5 (33.3%)	
- *Pseudomonas aeruginosa*	11 (16.9%)	2 (18.2%)	3 (27.3%)	3 (27.3%)	3 (27.3%)	
Total	60 (100%)	12 (20%)	18 (30%)	15 (25%)	15 (25%)	

ETT: endotracheal tube, BAL: bronchoalveolar lavage.

**Table 3:** Baseline molecular distribution of carbapenemase genes among CR-GNB isolates.

Carbapenem-Resistant Gram-Negative Isolates	Total Isolates *n* = 60	*bla*_NDM_ *n* = 35 (58.3%)	*bla*_OXA-48_ *n* = 25 (41.6%)	*bla*_KPC_ *n* = 24 (40%)	*bla*_VIM_ *n* = 22 (36.6%)	*bla*_IMP_ *n* = 29 (48.3%)
Fermenters	34					
- *Klebsiella pneumoniae*	25	15 (60%)	11 (44%)	9 (36%)	11 (44%)	14 (56%)
- *Escherichia coli*	7	0 (0%)	2 (28%)	2 (28%)	2 (28%)	2 (28%)
- *Enterobacter cloacae*	2	0 (0%)	0 (0%)	0 (0%)	1 (50%)	0 (0%)
Non-Fermenters	26					
- *Acinetobacter baumannii*	15	11 (73%)	9 (60%)	6 (40%)	4 (26%)	7 (46%)
- *Pseudomonas aeruginosa*	11	10 (90%)	3 (27%)	7 (63%)	4 (36%)	6 (54%)

**Table 4:** Comparative diagnostic performance of the direct-from-specimen LFIA stratified by pathogen type.

Carbapenem-Resistant Gram-Negative Isolates	Gene Target	Raw Outcomes (TP/FP/FN/TN)	Sensitivity % (95% CI)	Specificity % (95% CI)	Accuracy %	Cohen’s Kappa (κ) *
Fermenters						
*Klebsiella pneumoniae* (*n* = 25)						
	*bla*_NDM_	11/0/4/10	73.3%	100%	84.0%	0.687
	*bla*_OXA-48_	11/5/0/9	100%	64.3%	80.0%	0.613
	*bla*_KPC_	9/0/0/16	100%	100%	100%	1.000
	*bla*_VIM_	3/0/8/14	27.3%	100%	68.0%	0.296
	*bla*_IMP_	12/0/2/11	85.7%	100%	92.0%	0.841
*Escherichia* *coli* (*n* = 7)						
	*bla*_NDM_	0/0/0/7	N/A *	100%	100%	1.000
	*bla*_OXA-48_	2/0/0/5	100%	100%	100%	1.000
	*bla*_KPC_	0/0/2/5	0.0%	100%	71.4%	0.000
	*bla*_VIM_	0/0/2/5	0.0%	100%	71.4%	0.000
	*bla*_IMP_	0/0/2/5	0.0%	100%	71.4%	0.000
*Enterobacter cloacae* (*n* = 2)						
	*bla*_NDM_	0/0/0/2	N/A *	100%	100%	1.000
	*bla*_OXA-48_	0/0/0/2	N/A *	100%	100%	1.000
	*bla*_KPC_	0/0/0/2	N/A *	100%	100%	1.000
	*bla*_VIM_	0/0/1/1	0.0%	100%	50.0%	0.000
	*bla*_IMP_	0/0/0/2	N/A *	100%	100%	1.000
Non-fermenters						
*Acinetobacter baumannii* (*n* = 15)						
	*bla*_NDM_	11/1/0/3	100%	75.0%	93.3%	0.815
	*bla*_OXA-48_	9/0/0/6	100%	100%	100%	1.000
	*bla*_KPC_	6/1/0/8	100%	88.9%	93.3%	0.865
	*bla*_VIM_	0/0/4/11	0.0%	100%	73.3%	0.000
	*bla*_IMP_	7/0/0/8	100%	100%	100%	1.000
*Pseudomonas aeruginosa* (*n* = 11)						
	*bla*_NDM_	10/1/0/0	100%	0.0% **	90.9%	0.000 **
	*bla*_OXA-48_	3/1/0/7	100%	87.5%	90.9%	0.792
	*bla*_KPC_	5/0/2/4	71.4%	100%	81.8%	0.645
	*bla*_VIM_	1/0/3/7	25.0%	100%	72.7%	0.298
	*bla*_IMP_	5/0/1/5	83.3%	100%	90.9%	0.820

TP/FP/FN/TN (True Positive/False Positive/False Negative/True Negative), 95% Confidence Intervals (CI) were calculated using the Wilson score interval method. N/A indicates sensitivity could not be calculated due to a complete absence of the target gene in that specific sub-cohort. * Cohen’s Kappa (kappa) was computed to assess the categorical diagnostic agreement and inter-rater reliability between the PCR gold standard and the direct-from-specimen-LFIA. Kappa interpretation: < 0 (no agreement), 0.01–0.20 (none to slight), 0.21–0.40 (fair), 0.41–0.60 (moderate), 0.61–0.80 (considerable), and 0.81–1.00 (almost perfect agreement) [21]. ** The 0.0% specificity and 0.000 Kappa for NDM in *Pseudomonas aeruginosa* are mathematical artifacts due to the absence of any TN isolates in this sub-cohort (*n* = 11); all isolates either truly possessed the gene or triggered a false positive.

**Table 5:** Comparative diagnostic performance of the direct-from-specimen LFIA stratified by clinical matrices.

Clinical Matrix	Carbapenemase Genotype Target	PCR Positive N (%)	LFIA Positive N (%)	Sensitivity (95% CI)	Specificity (95% CI)	PPV	NPV	Accuracy	Cohen’s Kappa (κ)	*p*-Value
Blood (*n* = 12)	*bla*_NDM_	7 (58.3%)	6 (50%)	85.7% (42.1–99.6)	100% (47.8–100)	100% (59.8–100)	83.3% (35.9–99.6)	91.7%	0.833	0.003
	*bla*_OXA-48_	5 (41.7%)	5 (41.7%)	80.0% (28.4–99.5)	85.7% (42.1–99.6)	80.0% (28.4–99.5)	85.7% (42.1–99.6)	83.3%	0.657	0.023
	*bla*_KPC_	4 (33.3%)	3 (25%)	75.0% (19.4–99.4)	100% (63.1–100)	100% (47.8–100)	88.9% (51.8–99.7)	91.7%	0.800	0.005 **
	*bla*_VIM_	3 (25%)	0 (0%)	0% (0–70.8)	100% (66.4–100)	0.0% (0–100)	75.0% (39.0–94.0)	75%	-	-
	*bla*_IMP_	4 (33.3%)	3 (25%)	75.0% (19.4–99.4)	100% (63.1–100)	100% (47.8–100)	88.9% (51.8–99.7)	91.6%	0.800	0.005
Urine (*n* = 18)	*bla*_NDM_	7 (38.9%)	6 (33.3%)	85.7% (42.1–99.6)	100% (71.5–100)	100% (59.8–100)	91.7% (61.5–99.8)	94.4%	0.880	<0.001 **
	*bla*_OXA-48_	6 (33.3%)	7 (38.9%)	83.3% (35.9–99.6)	83.3% (35.9–99.6)	71.4% (29.0–96.3)	90.9% (58.7–99.8)	83.3%	0.640	0.006 **
	*bla*_KPC_	7 (38.9%)	5 (27.8%)	71.4% (29.0–96.3)	100% (71.5–100)	100% (59.0–100)	84.6% (54.6–98.1)	88.9%	0.753	<0.001 **
	*bla*_VIM_	6 (33.3%)	1 (5.6%)	16.7% (0.4–64.1)	100% (73.5–100)	100% (2.5–100)	70.6% (44.0–89.7)	72.2%	0.211	0.146
	*bla*_IMP_	10 (55.6%)	7 (38.9%)	70.0% (34.8–93.3)	100% (63.1–100)	100% (59.8–100)	72.7% (39.0–94.0)	83.33%	0.675	0.002 **
Respiratory (*n* = 30)	*bla*_NDM_	22 (62.9%)	22 (62.9%)	90.9% (70.8–98.9)	84.6% (54.6–98.1)	90.9% (70.8–98.9)	84.6% (54.6–98.1)	88.5%	0.755	<0.001 **
	*bla*_OXA-48_	14 (40%)	20 (57.1%)	85.7% (57.2–98.2)	61.9% (38.4–81.9)	60.0% (36.1–80.9)	86.7% (59.5–98.3)	71.4%	0.444	0.005 **
	*bla*_KPC_	13 (37.1%)	13 (37.1%)	84.6% (54.6–98.1)	90.9% (70.8–98.9)	84.6% (54.6–98.1)	90.9% (70.8–98.9)	88.6%	0.755	<0.001 **
	*bla*_VIM_	13 (37.1%)	3 (8.6%)	23.1% (5.0–53.8)	100% (84.6–100)	100% (29.2–100)	68.8% (50.0–83.9)	71.42%	0.274	0.018 *
	*bla*_IMP_	15 (42.9%)	14 (40%)	86.7% (59.5–98.3	95.0% (75.1–99.9)	92.9% (66.1–99.8)	90.5% (69.6–98.8)	91.42%	0.824	<0.001 **

* significant. ** highly significant.

**Table 6:** Comparative turnaround time (TAT) analysis of direct-from-specimen LFIA vs. culture-enhanced PCR, and standard phenotypic culture with VITEK 2 across clinical specimens.

Sample Matrix (*n* = 60)	Protocol	Mean TAT ± SD (min)	Median TAT (IQR) (min)	Range (Min–Max) (min)	*p*-Value LFIA vs. PCR	*p*-Value PCR vs. Routine Culture	*p*-Value LFIA vs. Routine Culture
Blood (*n* = 12)	Direct LFIA	48.58 ± 10.66	44 (22)	27 (36–63)	<0.001	<0.001	<0.001
	Culture-enhanced PCR	1740.83 ± 217.3	1766 (432)	650 (1453–2103)			
	Routine Culture +VITEK2 ID and AST	4293.83 ± 1087.6	4414 (1946)	2858 (2901–5759)			
Urine (*n* = 18)	Direct LFIA	52.56 ± 7.7	54.5 (13)	25 (37–62)	<0.001	<0.001	<0.001
	Culture-enhanced PCR	1813.9 ± 226.1	1803 (430)	705 (1448–2153)			
	Routine Culture, VITEK2 ID and AST	4012.5 ± 754.4	4059 (1369)	2570 (3013–5583)			
Respiratory (*n* = 30)	Direct LFIA	51.4 ± 9.35	52 (17)	29 (35–64)	<0.001	<0.001	<0.001
	Culture-enhanced PCR	1713.9 ± 170.1	1725 (260)	653 (1447–2100)			
	Routine Culture, VITEK2 ID and AST	4014.74 ± 837.3	3816 (1669)	2649 (2918–5567)			

TAT: turnaround time; min: minutes; ID: identification; AST: antimicrobial sensitivity; PCR: polymerase chain reaction.

## Data Availability

The associated author can provide the data upon request.

## Supplementary Materials

The following supporting information can be downloaded at: https://www.mdpi.com/article/10.3390/pathogens15060634/s1, Table S1: Primers utilized for the identification of the five major carbapenemase genes [18]; Figure S1: Kaplan–Meier curves illustrating time-to-results for three modalities (direct LFIA, multiplex PCR, and conventional culture).
