# Supplementary material for: Direct-from-Specimen Detection of Major Carbapenemases by Carbapenem-Resistant K.N.I.V.O. Detection K-Set: Comparative Analysis of Accuracy and Turnaround Time
Source: Pathogens. 2026 Jun 15;15(6):634. doi: 10.3390/pathogens15060634 (PMC13304714; doi:10.3390/pathogens15060634)
Supplement: Supplementary file 1 [file pathogens-15-00634-s001.zip › pathogens-4336314-supplementary.pdf]

Supplementary materials

Table S1. Primers Utilized for the Identification of the Five Major Carbapenemase Genes [18]

Target genes	Primer sequence	Amplicon size (pb)
bla$_{IMP}$	F: 5´- GGAATAGAGTGGCTTAAYTCTC-3´<br>R: 5´- GGTTTAAYAAAACAACCACC-3´	232
bla$_{KPC}$	F: 5´- CGTCTAGTTCTGCTGTCTTG-3´<br>R: 5´- CTTGTCATCCTTGTTAGGCG-3´	798
bla$_{NDM}$	F: 5´- GGTTTGGCGATCTGGTTTTC-3´<br>R: 5´- CGGAATGGCTCATCACGATC-3´	621
bla$_{OXA-48}$	F: 5´- GCGTGGTTAAGGATGAACAC-3´<br>R: 5´- CATCAAGTTCAACCCAACCG-3´	438
bla$_{VIM}$	F: 5´- GATGGTGTTTGGTCGCATA-3´<br>R: 5´- CGAATGCGCAGCACCAG-3´	390

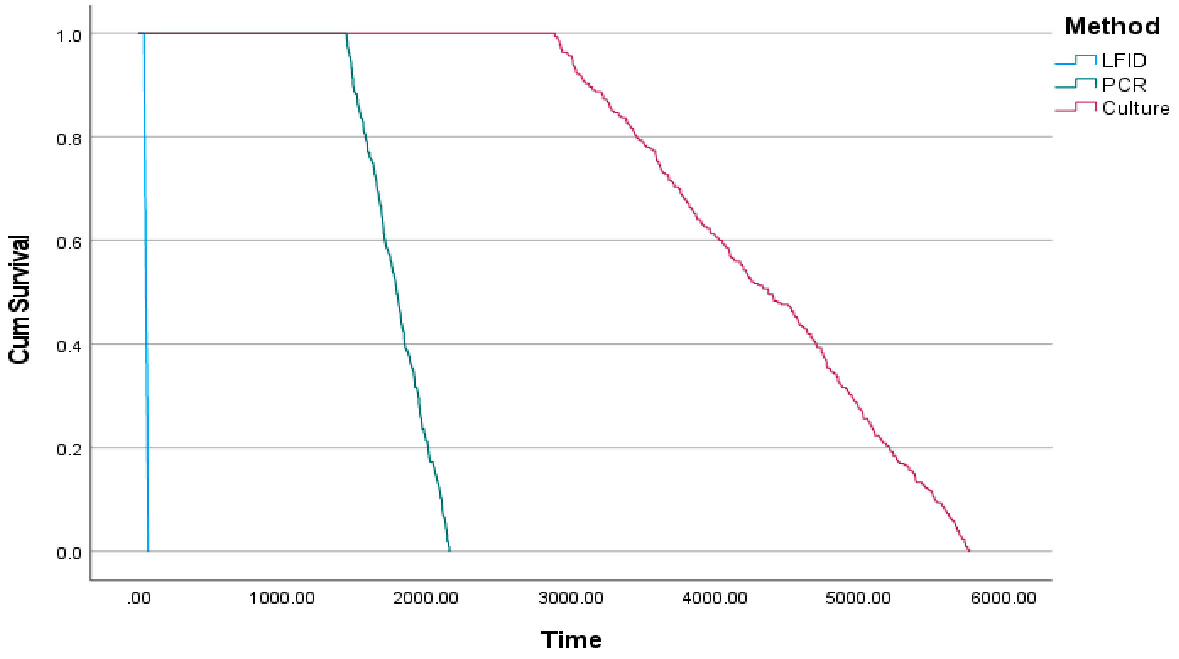

Figure S1. Kaplan–Meier curves illustrating time-to-results for three modalities (direct LFIA, multiplex PCR, and conventional culture). Direct LFIA demonstrated the fastest detection with a rapid decline in survival probability, followed by PCR, while culture exhibited the slowest detection rate.
